# Pattern recognition of topologically associating domains using deep learning

**DOI:** 10.1186/s12859-022-05075-1

**Published:** 2022-12-08

**Authors:** Jhen Yuan Yang, Jia-Ming Chang

**Affiliations:** Department of Computer Science, National Chengchi University, 11605 Taipei City, Taiwan

**Keywords:** Topologically associating domain, TAD, Hi-C, Chromosome organization, Deep learning

## Abstract

**Background:**

Recent increasing evidence indicates that three-dimensional chromosome structure plays an important role in genomic function. Topologically associating domains (TADs) are self-interacting regions that have been shown to be a chromosomal structural unit. During evolution, these are conserved based on checking synteny block cross species. Are there common TAD patterns across species or cell lines?

**Results:**

To address the above question, we propose a novel task—TAD recognition—as opposed to traditional TAD identification. Specifically, we treat Hi-C maps as images, thus re-casting TAD recognition as image pattern recognition, for which we use a convolutional neural network and a residual neural network. In addition, we propose an elegant way to generate non-TAD data for binary classification. We demonstrate deep learning performance which is quite promising, AUC > 0.80, through cross-species and cell-type validation.

**Conclusions:**

TADs have been shown to be conserved during evolution. Interestingly, our results confirm that the TAD recognition model is practical across species, which indicates that TADs between human and mouse show common patterns from an image classification point of view. Our approach could be a new way to identify TAD variations or patterns among Hi-C maps. For example, TADs of two Hi-C maps are conserved if the two classification models are exchangeable.

**Supplementary Information:**

The online version contains supplementary material available at 10.1186/s12859-022-05075-1.

## Introduction

Emerging evidence suggests that the structures of chromosomes and genome interactions are highly related [1]; deciphering the relationship between them will aid in our understanding of genetic function. Chromosome conformation capture (3C), which was developed to gain better insight into three-dimensional chromatin structures [2], requires a set of target gene loci, making chromatin-wide structures studies impossible. During the last decade, extensions to 3C such as 4C [3], 5C [4], and Hi-C [5] have been developed to determine spatial chromosomal interaction within a chromosome region, a chromosome, or an entire genome with unprecedented resolution and accuracy.

In 2009, Liebermann-Aiden proposed high-throughput chromosome conformation capture (Hi-C), which identifies long-range interactions in an unbiased, genome-wide fashion via high-throughput sequencing and bioinformatics analysis to understand the genome-wide relationship between chromosome organization and genome activity [5]; a resulting matrix of interaction frequencies shows the relative spatial disposition between two genomic regions in chromosome 3D space [5]. In short, Hi-C allows us to evaluate contact frequency between any pair of genomic loci.

### Topologically associating domains (TADs)

Recent studies on Hi-C data have demonstrated the existence of a type of chromatin structure known as a topologically associating domain (TAD) in which interactions are highly enriched inside each segment and are significantly depleted between adjacent segments; these can be seen as continuous square (blocks) domains of highly self-interacting regions along the diagonal of the Hi-C contact matrix [6, 7]. TADs are considered to be the structural and functional unit of a chromosome, at least in *Drosophila* and mammalian genomes [7, 8]. The functions of TADs are not fully understood, but some work shows that disturbing TAD boundaries affects the expression of nearby genes and is associated with disease [9].

TAD identification is an important problem for bioinformatics. In recent years, several different tools (TAD callers) have been made available for TAD detection from Hi-C. These can be divided into four categories: linear score, clustering, statistical model, and network features [10–12] (a list in Additional file 1: Table S1); however, it is not clear how to evaluate the accuracy of a TAD caller with no stable ground truth, given that TAD predictions vary significantly between different callers [10].

### Deep learning for image recognition

In 1980, Fukushima proposed the neocognitron [13], a hierarchical neural network (NN) used for tasks such as recognizing patterns by learning the shapes of the target objects. The neocognitron was inspired by simple and complex cells and is considered the prototype of the modern convolutional neural network (CNN). In 1998, Yann Lecun proposed the famous LeNet, the first CNN [14].

Jonathan Long proposed a fully convolutional neural network (FCN) for image segmentation [15] which performs end-to-end and pixel-to-pixel semantic segmentation. The final image segmentation is constructed from deconvolution based on upsampling.

A residual neural network (ResNet) is a CNN milestone, owing to its winning the first place in the ILSRVC competition and COCO contest in 2015 [16]. ResNet is outstanding when dealing with degradation because of its unique trick: the shortcut connection. It skips one or a few layers, utilizing residual mapping and adding this to the output of the stacking layers. Also, short connections require only the mapping to have the same shape as the output layers. This thus entails neither extra parameters nor extra computational complexity in the networks, such that nothing extra is required to train the residual network through the common backpropagation process.

A squeeze-and-excitation network (SENet) reinforces important features to increase prediction accuracy by modeling correlation between feature maps [17]. It won the first place in ILSRVC2017. Specifically, a SENet is merely the application of SE blocks in any neural network. A SE block consists of two major operations: *squeeze* and *excitation*. The squeeze operation shrinks the input feature maps through spatial dimensions via global average pooling (GAP) to generate smaller feature maps with overall information for each of their corresponding feature maps, after which the excitation operation calculates the weights of each feature map through extra fully connected layers and non-linear layers. We multiply these weights with the original feature maps to yield the final result.

Deep learning is gaining popularity for Hi-C data analysis. Henderson et al*.* achieved 96% accuracy in predicting TAD boundaries based on DNA sequences with three convolutional layers followed by a long short-term-memory layer [18]. Yan Zhang et al*.* applied a deep CNN to infer high-resolution Hi-C interaction matrices from low-resolution Hi-C data by considering the Hi-C map as an image [19].

### TAD recognition using deep learning

Although 3D chromatin is dynamic during cell differentiation [20] and even in daily life [21], the TAD boundary varies little [22, 23]. Dixon and colleagues [6] found the domain boundaries to be largely invariant between cell types when comparing mouse ES cells and cortex cells, or human ES cells and IMR90 cells. Regarding the evolution of chromosomal topology, they investigated TAD conservation between mouse and human and establish similar chromatin structure in syntenic regions where 53.8% of human boundaries are present in mouse boundaries and 75.9% of mouse boundaries in human boundaries. Going further, Rudan et al*.* observed the extensive conservation of chromosomal structure across four mammalian species (mouse, dog, rabbit, and macaque) [24].

It has been shown that CTCF binding is maintained at TAD boundaries [25]. Apart from genomic sequence patterns in TAD boundaries, are there any common TAD patterns across species or cell type? To address this question, we formalize a *TAD recognition* problem, determining whether a given genomic region is a TAD or not. In practice, could we build a TAD binary classification model of one species or one tissue, and then apply this to other species or tissue? If yes, we could conclude there is an exchange pattern across species or tissues.

Here, we are the first to consider TAD recognition as image recognition, i.e., predicting whether a given Hi-C region (an image) is a TAD or not (non-TAD). To increase feature maps, we use the SE-FCN and SE-ResNet (FCN and ResNet with SE blocks, respectively) deep learning models. Based on cross-species evaluation, prediction accuracy is reasonably high (~ 80%).

## Results

### Five-fold cross validation with a species-specific dataset

We performed five-fold cross validation using species-specific datasets _Human and _Mouse to evaluate the performance of the two deep learning methods. Table 1 shows that SE-ResNet is competitive with SE-FCN on both datasets. Interestingly, the performance of SE-ResNet is also more stable than that of SE-FCN across the five folds (detailed information on the individual folds are provided in Additional file 1: Tables S2–S3 for Human and S4–S5 for Mouse, respectively). Then, we validated the effect of label imbalance using three different ratios between TAD (positive) and non-TAD (negative). SE-ResNet shows steadier performance on the human set (0.89–0.91) compared with the mouse set (0.79–0.86) across evaluation metrics (complete metrics in Additional file 1: Tables S2–S5). More specifically, the ratio-1:1 model exhibited the least metric variation. Clearly, the more imbalanced the data is, the more challenging it is to train the model. Thus, SE-ResNet based on 1:1 (TAD:non-TAD) is used in the following experiments.

**Table 1:** Average of five-fold cross validation of deep learning models in Human and Mouse Hi-C datasets given various TAD: non-TAD ratios

Models	1:1			1:1.5			1:2		
	Acc.	F1	AUC	Acc.	F1	AUC	Acc.	F1	AUC
_Human									
SE-FCN	0.892	0.894	0.889	0.914	0.895	0.914	0.915	0.872	0.905
SE-ResNet	0.877	0.869	0.877	0.897	0.867	0.888	0.898	0.835	0.873
_Mouse									
SE-FCN	0.836	0.825	0.836	0.857	0.809	0.846	0.879	0.817	0.866
SE-ResNet	0.868	0.853	0.856	0.883	0﻿.856	0.883	0.880	0.812	0.858

### Cross cell line, cross-species test

We validated the generalization ability of SE-ResNet across species and cell types in the following three different experiments (Table 2, detailed confusion matrix in Additional file 1: Table S6).

**Table 2:** SE-ResNet performance for three different experimental settings

Methods	TPR	TNR	Precision	Acc.	F1	AUC
Same species, cross cell types						
SE-ResNet*_*Human.ES	0.922	0.832	0.846	0.877	0.882	0.877
SE-ResNet_Mouse.ES	0.853	0.763	0.783	0.808	0.817	0.808
Cross cell types						
SE-ResNet_ES	0.907	0.841	0.851	0.874	0.878	0.874
Cross species						
SE-ResNet_Human	0.876	0.864	0.862	0.870	0.869	0.870
SE-ResNet_Mouse	0.837	0.900	0.893	0.868	0.864	0.868

First, SE-ResNet was trained on ES cells and tested on other cell types for the same species, i.e., _Human.ES was trained on human ES cells and tested on human hIMR cells; _Mouse.ES was trained on mouse ES cells and tested on mouse mCO cells. All _Human.ES metrics are higher than those of _Mouse.ES (Same species, cross cell types in Table 2). This indicates that mouse data is more heterogeneous than human data; that is, models trained on ES cells are less efficient in classifying TAD of mCO cell lines.

Following the above test, we attempted to increase the training power for ES cells by merging human and mouse data into one set. Thus, we trained the _ES model on both human and mouse ES cells and tested it on human hIMR and mouse mCO cells. The resultant _ES performance was as expected, falling between _Human.ES and _Mouse.ES, except for TNR and precision (Cross cell types, Table 2). This shows that increasing the training data yields improved TAD classification for mCO but decreases the specificity of hIMR TADs.

We then validated the effectiveness of SE-ResNet by a cross-species test. We trained a model on one species and tested it on other species, i.e., _Human trained in human ES and hIMR cells and tested on mouse ES and Cortex cells. On average, the model achieved reasonable performance, exceeding 0.80 for all metrics (Cross species, Table 2). TAD has been shown to be conserved across species [6]. Here, we confirm that the classification power of TAD transfers across species.

In sum, we evaluated the generalization power of the TAD classification model from different angles. The results show that model performance is affected little by the training species or cell lines. We are confident the proposed model will prove useful in other Hi-C datasets. Further, our approach could be a new way to test TAD variations or patterns among Hi-C maps. For example, TADs of two Hi-C maps are conserved if two classification models are exchangeable.

## Discussion

### TAD patterns

CNN-based approaches for classification are becoming more popular. However, despite their strong performance, their training processes are difficult to unravel. We know only the inputs and outputs; everything else is a black box. In 2015, class activation mapping (CAM) was proposed by Zhou et al. [26]. The concept of CAM is intuitive: connect the GAP layer before the final classifier and sum all the feature vectors, multiplying by their corresponding weights given the specified label. This reveals which part of the image the model is focusing on [26].

**Fig. 1:**
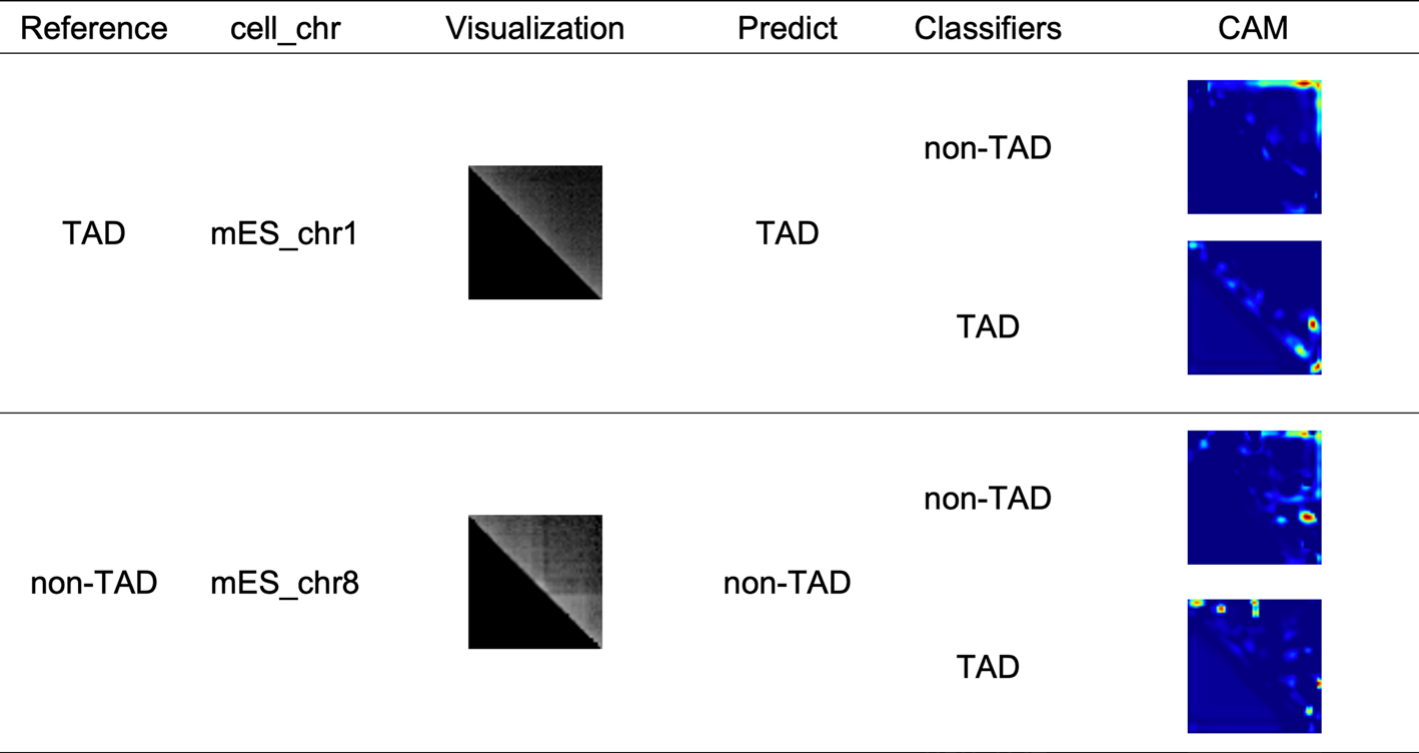
CAM of SE-ResNet_Human model applied on mES_chr1 and mES_chr8, where the model emphasizes and ignores parts colored in red and blue, respectively

CAM affords a clearer look at how the trained models classify a given input, indirectly proving that CNNs can localize a given discriminative pattern. In Fig. 1, we visualize the focused parts of SE-ResNet_Human on two cross-species testing cases. The first case, mES_chr1, is a TAD and the model predicts it as such. The next, mES_chr8, is non-TAD and the model also makes a correct prediction. CAM visualization of the two classifiers shows different discriminative patterns. Non-TAD focuses on the middle part whereas TAD focuses on the two ends of the region, which might be related to the enrichment of the CTCF binding site around TAD boundary [25, 27]. Whether TAD or non-TAD is predicted depends on which corresponding classifier output has a higher softmax probability.

### TAD identification

We have successfully classified TAD and non-TAD, which has not been addressed before. We extend a classification step to TAD identification. We develop a TAD caller, TADL, based on dynamic programming, which involves solving a problem by breaking it down into small easy sub-problems. The solution of the original problem is then based on the solutions of the sub-problems. That is, we simplify the complicated TAD identification of the whole genome by breaking it down into TAD recognition of specific regions in a recursive manner via dynamic programming. The algorithm for the TADL is provided in the following.
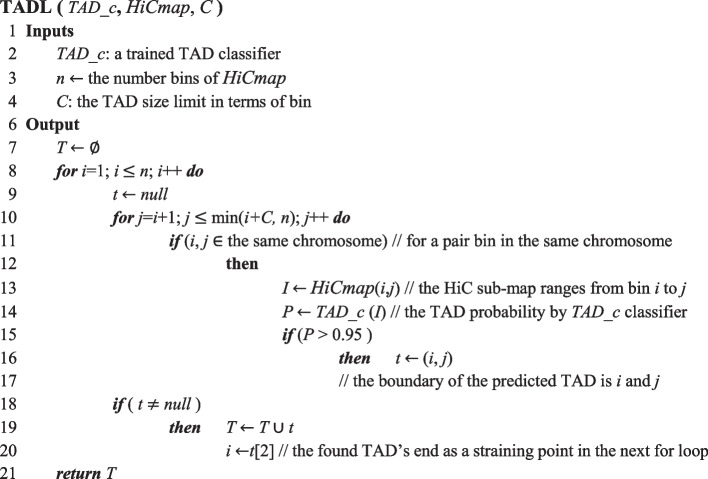


We start from the beginning of the Hi-C map and extend one bin (line 8 and 10). Then the region is classified as TAD or non-TAD using the previous trained model (line 14). If the TAD prediction probability of the region is more than 0.95, its beginning and end positions are saved (line 16). Otherwise, we enlarge the region one bin more within TAD size limit (line 10) and repeat the above classification step (line 14). After the enlarging step, if there is a saved region (line 18), the last saved one is returned as one TAD (line 19) and its end position is then taken as the starting point for the next process (line 20).

**Fig. 2:**
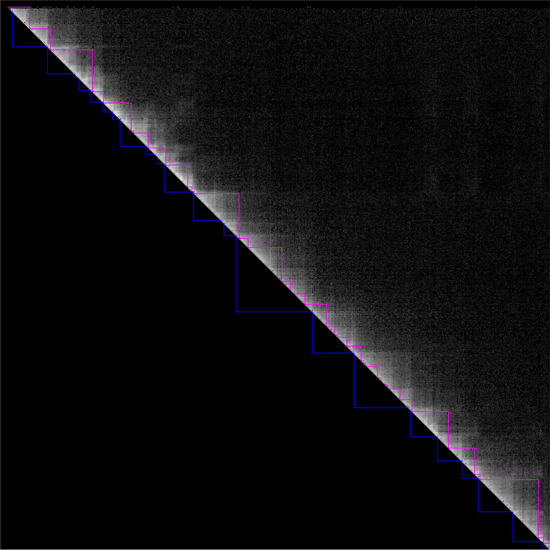
TAD caller demonstrated on mES chromosome 15:0..32000000. Lower blue triangles are TADs identified by *directionality index*, and upper pink triangles are TADs identified by TADL, SE-ResNet_Human

Here, Fig. 2 demonstrates the performance of the proposed TAD caller in a cross-species test in which TADs of mES Hi-C identified based on the SE-ResNet_Human TAD classifier, TADL(SE-ResNet_Human, mES Hi-C, 11), (top-right) are compared with those identified by the *direction index* (DI) [6] (bottom-left). Although they are not identical, most are consistent with each other, falling in Case A or B in Fig. 7a [28].

It has been shown that TAD boundaries have an enrichment of CTCF binding sites [25, 29]. We take mouse cortex and mESC CTCF ChIP-Seq [30] as an evaluation criterion to quantify the number of called TAD boundaries overlapped with CTCF peaks. The peak position is binned at a 40 kb resolution to ensure consistency with the Hi-C map resolution used here. This yields 22,535 and 22,505 CTCF peaks of mES and mCO, respectively. We benchmarked the performance of the SE-RestNet_Human TADL classifier on the mES and mCO datasets with DI and ClusterTAD [28], which is consistent and complementary with existing methods. The TAD boundary of DI is from publicly available data [31]. We run ClusterTAD locally with default parameters at a 40 kb resolution and use the TADs defined in BestTAD_*.txt file.

Table 3 summarizes the results of TAD and CTCF evaluation. The proposed method tends to generate more and smaller TADs compared with DI and ClusterTAD. ClusterTAD detects fewer TADs than originally reported [31] due to the larger Hi-C bin size used here. Although DI detects fewer TADs, most match accurately with the CTCF peaks (higher precision). Interestingly, if we relax the overlapping peaks within the TAD boundary interval ± 1 bin size (“Extended” in Table 3), our method detects far more TADs matching CTCF points than DI (higher recall). ClusterTAD is the most precise method in the extended metrics.

**Table 3:** TADs of mES and mCO Hi-C using DI, ClusterTAD, and TADL SE-RestNet_Human; the corresponding evaluation, the number of CTCF peaks overlapping the TAD boundary based on original boundary and extended positions

Method	TAD		# of overlapping CTCF	
	Number	Size (bin)	Original	Extended
mES				
ClusterTAD	1224	46.91	1565	3883
DI	2200	27.54	2951	3981
TADL	3732	15.15	2849	4986
mCO				
ClusterTAD	1226	46.22	1500	3887
DI	1519	37.67	1970	2691
TADL	3390	14.68	2695	4765

## Conclusion

We are the first to propose TAD classification as image classification, where Hi-C data is re-cast as an image. We explore deep learning methods including CNN, ResNet, and SENet to classify TADs from sub-kb high-resolution chromatin capture experiments. To train a binary classification model, we generate negative cases (non-TAD). The usefulness of the model is validated through cross-species tests. Model accuracies exceed 80% and the other metrics are also decent. Further, some are extremely close to the accuracy metric, indicating that the model’s performance is not biased toward a specific class, TAD or non-TAD. Deep learning technology has progressed rapidly for image classification. By re-framing the problem as image classification, TAD classification performance will continue to improve as deep learning progresses. TAD has shown to be conserved during evolution. Interestingly, our results confirm that the TAD classification model is practical across species. This indicates TADs between human and mouse show common patterns from an image classification perspective. Our approach could be a new way to test TAD variations or patterns in Hi-C maps. For example, TADs of two Hi-C maps are conserved if two classification models are exchangeable.

Our model was trained using a Hi-C contact matrix at a 40 kb bin-size resolution. It would be instructive to evaluate the 40 kb model capacity in other resolutions, in particular higher resolutions, e.g., 1 kb bin-size, which are currently available for many species [20, 29, 32]. Alternatively, the same deep learning architecture could be further re-trained using higher-resolution Hi-C maps.

## Methods

### Hi-C data preparation

The Hi-C dataset we used consists of mouse embryonic stem cell (mESC) and mouse cortex (mCO) mouse types as well as human embryonic stem cell (hESC) and human IMR90 fibroblasts (hIMR) human types. All interaction matrices were created using a 40 kb bin size throughout the genome. The Hi-C dataset was already normalized using an integrated probabilistic background model [33] to estimate its parameters and renormalize the Hi-C data (downloaded from [34] with 40 kb resolution).

The Hi-C data is a contact matrix featuring spatial chromosomal interaction within a chromosome region. Because the Hi-C contact matrix is symmetric, only the contacts in the upper triangle along the diagonal of the contact matrix were considered. We added 1 to all elements in the matrix to avoid 0 elements, and then applied log2 for transformation purposes. Dividing the whole contact matrix by the maximum element yielded a matrix whose element values ranged from 0 to 1.

We also knew the locations of each of the TADs that we called in the Hi-C data (downloaded from [34]). The TADs for each dataset were provided as bed files, with each line containing three types of TAD information: chromosome, domain start, and domain end. As the TADs were called using the normalized data with a bin size of 40 kb, each start and end position was a multiple of 40 k.

#### Non-TAD generation

For training in the TAD binary classification problem, we collected positive (TAD) and negative (non-TAD) data. Generally, TAD callers search only for TADs in Hi-C contact matrices and do not provide non-TAD instances. Hence, we generated a non-TAD set from the Hi-C contact matrix.

**Fig. 3:**
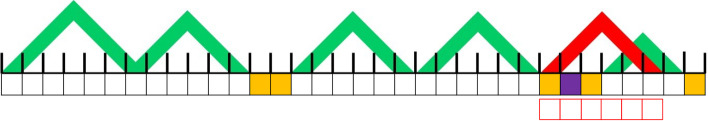
Generation of non-TADs. Orange squares are non-TAD points; a purple square is a randomly selected anchor point; the number of red squares corresponds a random TAD size; green triangles are TADs, and a red triangle is a non-TAD instance resulting from this procedure

First, we defined regions that do not belong to TAD domains as non-TAD points (Fig. 3, orange squares). To ensure a similar size distribution between TAD and non-TAD sets, one TAD was randomly selected among all TADs and its size was used to generate a non-TAD instance (e.g., size = 6). Then, we randomly chose one non-TAD point as an anchor point (Fig. 3, the purple square). Finally, a non-TAD (Fig. 3, the red triangle) was generated by arbitrarily mapping a selected rectangle (Fig. 3, red squares) to the anchor point. To evaluate the capacity of the model with imbalanced labels, we repeated the above process to generate three different amounts of non-TAD sets. As the model should be able to identify positive cases among noisy negative cases, three upsampled negative sets were constructed with positive-to-negative ratios of 1:1, 1:1.5, and 1:2 (Table 4).

**Table 4:** Numbers of TADs and non-TADs given different upsampling ratios

Type (ratio)	Mouse ES	Mouse cortex	Human ES	Human IMR90
TAD	2200	1519	3127	2349
Non-TAD				
1.0	2200	1519	3127	2349
1.5	3292	2273	4684	3517
2.0	4400	3038	6254	4698

#### HiC data transformation to image

Both TAD and non-TAD datasets were resized to 60X60 and saved as image files using the *opencv* library’s *cv2.resize* and *cv2.imwrite* functions, respectively. The image files are publicly available at https://doi.org/10.6084/m9.figshare.9697451. The final dimension of our data was 60 × 60 × 3, where 3 corresponds to the RGB channels.

### Deep learning models

We evaluated two CNN models: a fully convolutional network (FCN) and a residual neural network model (ResNet). We added to both models squeeze-and-excitation (SE) blocks to increase feature maps, and named these the SE-FCN and SE-ResNet models, respectively. We implemented the models in *Keras*, a high-level neural network API written in Python which runs on top of TensorFlow, CNTK, or Theano.

#### Model architectures

**Fig. 4:**
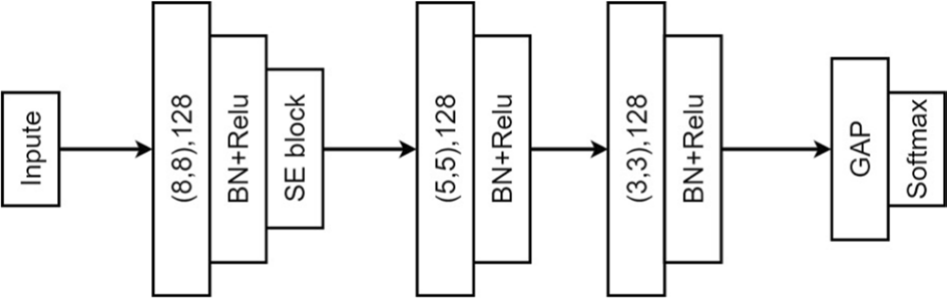
SE-FCN model combined with three blocks, where (8, 8), (5, 5), and (3, 3) correspond to the filter size and 128 is the kernel size

We first explored the SE-FCN model, which is based on squeeze-and-excitation and fully convolutional networks (Fig. 4). Filter sizes 8, 5, and 3 are labeled on the convolutional block from left to right with the same kernel size, 128. The output of the convolution layer passes through a batch normalization (BN) layer (*BatchNormalization* layer, Keras) with ReLU activations. We use BN to mitigate internal covariate shift [35]. To intensify features, only the first block has a SE block. Finally, the result flows into the GAP layer with a softmax layer. For binary classification, softmax is applied to the output to determine which of TAD or non-TAD is more reliable; this yields a set of probabilities.

**Fig. 5:**
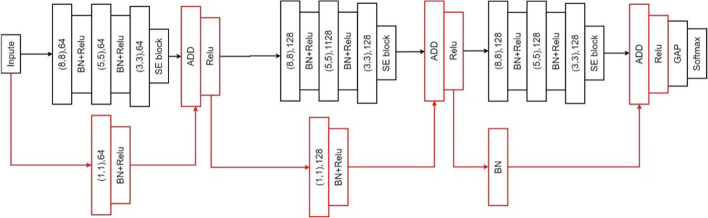
SE-ResNet model, combined with three SE-FCN blocks and additional components marked in red

SE-ResNet, the second model, is composed of a series of SE-FCN blocks, the difference being that the SE block is placed after convolutional blocks with filter sizes of 3 [26]. Also, the input values are regulated by the kernels and added to the output of the SE blocks. An operation that directly adds the input to the output of the following block is called a shortcut (Fig. 5).

### Evaluation

#### Experimental design

To estimate the performance of the deep learning models, we conducted experiments in two phases: training and independent test.

- Training: We applied five-fold cross validation to estimate the model’s performance on new data. The dataset was split into two non-overlapping sets, training and validation, at a 4:1 ratio. The former was used to train the weights of the model, and the latter was used to evaluate the predictive ability of the trained model. We used the Adam optimizer to minimize binary cross-entropy loss. The training period was set to 400 epochs and the batch size to 48 using the *KFold* function from the *Sklearn* package.
- Independent test: As a Hi-C map is an abstract representation of 3D chromosome structure, a cell type should contain its own structure pattern such that its Hi-C map might be different from those of other cell types. For a trained model, it is interesting to test the upper bound of classification power on Hi-C maps of other types. We performed the following experiments to train the model on one type and independently test it on a different type (Table 5).
  - Same species, Cross cell types
    - Human.ES: trained on human ES cell and tested on human IMR90 cell
    - Mouse.ES: trained on mouse ES cell and tested on mouse cortex cell
  - Cross cell types (ES): trained on both human and mouse ES cells and tested on human IMR90 and mouse cortex cells
  - Cross species
    - Human: trained on human ES and IMR90 cells and tested on mouse ES and cortex cells
    - Mouse: trained on mouse ES and cortex cells and tested on human ES and IMR90 cells

**Table 5:** Training and test data used in independent test phase

Label*	Mouse ES	Mouse cortex	Human ES	Human IMR90
Same species, cross cell types				
Human.ES	–	–	Train	Test
Mouse.ES	Train	Test	–	–
Cross cell types				
ES	Train	Test	Train	Test
Cross species				
Human	Test	Test	Train	Train
Mouse	Train	Train	Test	Test

*A label is added to the deep learning models as a suffix to indicate which experimental design was used. For example, SE-ResNet_Human indicates a SE-ResNet model trained on Human data and tested on Mouse data

#### Metrics

We used six different metrics to evaluate the performance of the proposed models from different perspectives: precision, recall (true positive rate, TPR), true negative rate (TNR), accuracy (Acc.), F1 score, and area under the curve (AUC). Precision is the fraction of the corresponding TADs among the retrieved TADs:

$${\text{Precision }} = TP/\left( {TP + FP} \right).$$

The relative frequencies of the correct results obtained on the set of all instances are defined as TPR (recall) and TNR, which are shown as

$$\begin{aligned} {\text{TPR }} & = TP/\left( {TP + FN} \right) \\ {\text{TNR}} & = TN/\left( {TN + FP} \right). \\ \end{aligned}$$

Accuracy (Acc.) can be seen as the total correct number of classifications, formulated as

$${\text{Acc. }} = \, \left( {TP + TN} \right)/\left( {TP + FP + TN + FN} \right).$$

The F1 score is the harmonic average of precision and recall. The F1 score is usually better than accuracy, especially given an uneven class distribution.

$${\text{F1 }} = { 2}*Recall*Precision/\left( {Recall + Precision} \right)$$

AUC is a performance measurement for classification problems at various threshold settings. Briefly, it indicates how well the model distinguishes between classes. A good model has an AUC close to 1, indicating good separation between positive and negative labels. In contrast, a poor model has an AUC near to 0, indicating poor separation between labels.

Because Accuracy does not take into account a model’s differential performance between two classes, metrics such as precision, TPR, TNR, and the F1 score provide supplemental information on whether the model performance is biased toward a specific class. AUC generally shows how a model performs across different thresholds applied to the probability of prediction. The model is considered reliable if the six metrics are stable on the validation set.

## Supplementary Information

**Additional file 1.** Additional results in **Tables S1–S6**.

## Data Availability

The materials generated and analyzed during the current study are available at: TAD and non-TAD image data used to train model https://doi.org/10.6084/m9.figshare.9697451.

## Abbreviations

TAD: Topologically associating domain
CNN: Convolutional neural network
FCN: Fully convolutional network
ResNet: Residual neural network
SE: Squeeze-and-excitation
GAP: Global average pooling
TPR: True positive rate
TNR: True negative rate
